# Intranasal Administration of Mesenchymal Stem Cell-Derived Exosome Alleviates Hypoxic-Ischemic Brain Injury

**DOI:** 10.3390/pharmaceutics16040446

**Published:** 2024-03-23

**Authors:** Takuma Ikeda, Masahito Kawabori, Yuyuan Zheng, Sho Yamaguchi, Shuho Gotoh, Yo Nakahara, Erika Yoshie, Miki Fujimura

**Affiliations:** 1 Department of Neurosurgery, Hokkaido University Graduate School of Medicine, Sapporo 060-8638, Hokkaido, Japan; ikeda.takuma.l2@elms.hokudai.ac.jp (T.I.); zhengyuyuan0214@outlook.com (Y.Z.); churabeaver@gmail.com (S.G.); yo.nakahara1014@gmail.com (Y.N.); yoshieri1124@gmail.com (E.Y.); fujimur@med.hokudai.ac.jp (M.F.); 2 Regenerative Medicine and Cell Therapy Laboratories, Kaneka, Kobe 650-0047, Hyogo, Japan; sho.yamaguchi1@kaneka.co.jp

**Keywords:** exosome, mesenchymal stem cell, intranasal administration, hypoxic-ischemic brain injury, inflammation

## Abstract

Hypoxic-ischemic brain injury arises from inadequate oxygen delivery to the brain, commonly occurring following cardiac arrest, which lacks effective treatments. Recent studies have demonstrated the therapeutic potential of exosomes released from mesenchymal stem cells. Given the challenge of systemic dilution associated with intravenous administration, intranasal delivery has emerged as a promising approach. In this study, we investigate the effects of intranasally administered exosomes in an animal model. Exosomes were isolated from the cell supernatants using the ultracentrifugation method. Brain injury was induced in Sprague-Dawley rats through a transient four-vessel occlusion model. Intranasal administration was conducted with 3 × 10^8^ exosome particles in 20 µL of PBS or PBS alone, administered daily for 7 days post-injury. Long-term cognitive behavioral assessments, biodistribution of exosomes, and histological evaluations of apoptosis and neuroinflammation were conducted. Exosomes were primarily detected in the olfactory bulb one hour after intranasal administration, subsequently distributing to the striatum and midbrain. Rats treated with exosomes exhibited substantial improvement in cognitive function up to 28 days after the insult, and demonstrated significantly fewer apoptotic cells along with higher neuronal cell survival in the hippocampus. Exosomes were found to be taken up by microglia, leading to a decrease in the expression of cytotoxic inflammatory markers.

## 1. Introduction

Hypoxic-ischemic brain injury (HIBI) occurs due to inadequate oxygenated blood flow to the brain. When the oxygen supply is significantly diminished, brain cells undergo damage, leading to long-term neurological consequences or even death. HIBI can manifest in various medical conditions, including cardiac arrest, drowning, severe respiratory distress, and choking. Annually, an estimated 360,000 to 550,000 individuals in the United States experience HIBI following cardiac arrest [1]. Currently, hypothermia therapy stands as the sole validated treatment for cardiac arrest patients; however, its efficacy is limited, with a survival rate of only 20–30% and a return-to-work rate of 9% [1,2,3]. Consequently, there exists a compelling need for innovative treatment strategies. Stem cell therapy has emerged as a promising therapeutic strategy for HIBI owing to its potential neurorestorative and immunomodulatory properties [4]. Recent advancements in stem cell research have revealed that one of the important therapeutic mechanisms of stem cell treatment is the exosome. Exosomes are nano-sized vesicles (40–200 nm), characterized by a double lipid layer membrane. They contain various bioactive molecules, including DNA, mRNA, microRNA (miRNA), and proteins, which are transferred to target cells to offer therapeutic benefits [5,6,7,8]. Their remarkable cryopreservation capacity and low immunogenicity have prompted considerations of mesenchymal stem cell (MSC)-derived exosomes as a promising alternative to MSC therapy. However, both exosomes and stem cells face challenges with systemic dilution when administered intravenously [9,10]. In these circumstances, intranasal administration is gaining considerable attention due to its convenient and efficient method of delivering exosomes to the brain [11]. Exosomes, when transplanted through the intranasal route, have been reported to be transferred into the brain through the olfactory and trigeminal nerves, mitigating neurological diseases through various therapeutic mechanisms, including inflammatory modulation. However, the efficacy of this approach has not been evaluated in models of HIBI [11]. Therefore, the objective of this study is to investigate whether intranasal administration of mesenchymal stem cell-derived exosomes can alleviate HIBI and elucidate the underlying therapeutic mechanisms.

## 2. Methods

Animal protocols were approved by the Animal Studies Ethics Committee of the Hokkaido University Graduate School of Medicine (approval number: 17-0065). All experimental procedures were conducted following the Institutional Guidelines for Animal Experimentation and the Guidelines for Proper Conduct of Animal Experiments by the Science Council of Japan.

### 2.1. Stem Cell Preparation and Exosome Extraction

Human adipose-derived mesenchymal stem cell (AMSC) vials were provided by Kaneka (Osaka, Japan) [12]. The cells were thawed and seeded at a concentration of 1.4 × 10^4^ cells/mL in medium for 3 days, as previously reported [12]. The medium was further replaced with serum-free cell culture medium (MEMα, 12561056, Thermo Fisher Scientific, Waltham, MA, USA) for 48 h to obtain exosome-containing medium supernatant. Ultra-centrifugation methods were utilized to isolate exosomes, as previously described with minor modification [13,14]. Briefly, the supernatant was continuously centrifugated at 2000× *g* for 10 min and 10,000× *g* for 30 min to remove dead cells and cellular debris. Subsequently, the supernatant was ultra-centrifugated at 100,000× *g* for 70 min, which forms a pellet consisting of exosomes. The morphology and size of the exosomes were confirmed through transmission electron microscopy (TEM, H-7100, Hitachi, Tokyo, Japan) and nanoparticle tracking analyzer (Videodrop, Myriade, Paris, France). Western blotting was then performed to confirm the presence of specific exosome surface markers, including CD9 (1:500, 014-27763, Fujifilm, Wako, Japan), CD63 (1:500, 012-27063, Fujifilm, Wako, Japan), and Calnexin (1:500, ab22595, Abcam, Cambridge, UK).

### 2.2. Animals Models of HIBI and Exosome Administration

Eight-week-old male Sprague-Dawley rats (CLEA Japan, Tokyo, Japan) weighing 250–300 g were used to create HIBI, as previously described with minor modification [15]. Briefly, after inducting anesthesia, bilateral vertebral arteries were dorsally exposed at C2 level, and were coagulated and cut. Twenty-four hours later, bilateral common carotid arteries (CCA) were exposed ventrally, and were temporary occluded for 20 min using vascular clip (JAN 0883475951578, Charmant, Toyama, Japan). After recanalization of the CCA, rats were returned to the cage. All Surgical procedures were conducted under anesthesia by 2–5% isoflurane in 30% O_2_ and 70% N_2_O and the body temperature of experimental animals was maintained at between 36.5 and 37.5 °C throughout the procedures using a heat pad. The death ratio during the surgery was 31.8%, and the animals that survived until the next day were used for the subsequent experiment. Rats that showed over a 25% decrease in body weight at day 3 were excluded and euthanized in consideration of animal welfare.

### 2.3. Intranasal Administration of Exosomes

Exosome administration was conducted as previously described with minor modification [16]. Animals were randomly assigned to one of two groups; the exosome group or the PBS group. Soon after transient CCA occlusion, the nasal airways were cleansed by hyaluronidase (100 U/5 μL each nostril) 30 min prior to exosome or PBS administration. This would allow clearing the nasal airway for better absorbance of exosomes. Subsequently, the exosome group received 3 × 10^8^ particles of exosomes in 20 µL of PBS, while the PBS group received 20 µL of PBS alone. Ten µL of exosomes or PBS was intranasally administered to each nostril using a micropipette equipped with a 10 µL pipette-tip. The animal was then placed in a dorsal position for 10 min to facilitate exosome absorption into the olfactory bulb. Exosomes were administered daily for up to seven days following the insult.

### 2.4. Neurological Assessments

The eight-arm radial maze test were conducted as previously reported [17]. Briefly, rats underwent food restriction to gradually reach 85% of their free-feeding weight over 5 training days (from day 5 to day 9 post HIBI insult). During this period, the rats were acclimated to the eight-arm radial maze (RR-10, Shinfactory, Fukuoka, Japan), where food pellets (45 mg BioServ, Frenchtown, NJ, USA) were positioned at the end of each arm as a reward. In each training trial, rats were released into the center of the maze and given 10 min to explore and collect pellets from all arms. Following the training period (from day 10 to day 15), pellets were randomly placed in three arms assigned for each animal, and the time taken and errors made to collect all pellets within 3 min were recorded. To eliminate the possibility of using internal cues such as residual smells, the maze was rotated by 90° daily, while maintaining the spatial location of the baited arms in relation to the room cues constant. The pellet locations remained consistent throughout the experiment, and three consecutive trials were conducted each day. The assessed behaviors included accuracy rate (number of entries into correct arm/number of entries into all arms), total distance moved, reference memory errors (entries into incorrect arms), and working memory errors (entries into arms pellets were already collected). The same tests were repeated two weeks after the last trial (on day 28) to assess long-term memory impairment.

### 2.5. Exosome Absorption and Distribution

Labeling of exosomes was performed with ExoSparkler Exosome Membrane Labeling Kit-Green (Dojindo, Kumamoto, Japan) following the manufacturer’s instructions. Following intranasal administration of exosomes, animals were sacrificed at 1, 3, and 24 h post-administration. Rats were deeply anesthetized and transcardially perfused with PBS followed by 4% paraformaldehyde (PFA), as previously reported [18]. The brains were then extracted, and fixed in 4% PFA for 24 h, embedded in paraffin, and sectioned into coronal slices of 4 μm in thickness. Serial sections of the olfactory nerve (15 mm anterior to the bregma), olfactory tract (5 mm anterior to the bregma), striatum (0 mm anterior to the bregma), and midbrain (7 mm posterior to the bregma) were prepared for the biodistribution analysis of labeled exosomes, with four non-overlapping regions of interest (ROI) set for each section. Positive fluorescence signals indicative of labeled exosomes were quantified using an automated cell/area counter according to the manufacturer’s instruction (BZ-X Analyzer, Keyence Co., Osaka, Japan). To eliminate autofluorescence signals in each area, the average fluorescence observed in the control group for each area was subtracted from the specimen.

### 2.6. Immunohistochemistry

Immunohistochemistry was conducted to assess neuronal damage in the hippocampus. To evaluate the potential of exosomes in attenuating neuronal apoptosis and neurodegeneration, TUNEL staining (ApopTag Fluorescein In Situ Apoptosis Detection Kit, S7110, Merck, Darmstadt, Germany) and Fluoro-Jade C staining (Biosensis, South Australia, Australia) were performed on day 3 following the manufacturer’s protocol [19,20,21]. Neuronal integrity was assessed using Nissl staining (0.1% Cresyl violet solution; Muto pure chemical, Tokyo, Japan) during the acute phase (day 3) and chronic phase (day 28). To evaluate the effect of exosomes on activated microglia/macrophages, Iba1 staining was performed (1:1500, 019-19741, Wako, Japan). Neuronal inflammation was assessed using IL-1β (1:500, ab283818; Abcam, Cambridge, UK), IL-6 (1:200, bs-0379R; Bioss Inc., Woburn, MA, USA), and TNF-α (1:1000, ab307164; Abcam, Cambridge, UK) staining for day 7 section. Five non-overlapping ROIs were designated in the hippocampal area. The number of positive signals, area, or luminescence was measured using an automated cell/area counter (BZ-X Analyzer, Keyence Co., Osaka, Japan).

### 2.7. In Vitro Microglial Assay

Microglial inflammatory assay was conducted as previously described [22]. Briefly, mouse BV2 microglia were obtained from Elabscience (Houston, TX, USA) and cultured in MEMα with 10% FBS and 1% penicillin/streptomycin. BV2 microglia cells (2 × 10^4^ cells/well) were seeded in a 96-well plate with MEMα. Twenty-four hours after seeding, BV2 cells were stimulated with 10 μg/mL of lipopolysaccharides (LPS) for assessing cell viability, and stimulated with 0.1 μg/mL of LPS for IL-6 and TNF-α production. Subsequently, they were co-cultured with exosomes (50 μg/mL) for 24 h. Cell viability was determined using a CCK-8 kit (Dojindo Laboratories, Kumamoto, Japan), and the levels of IL-6 and TNF-α were measured in the supernatant using enzyme-linked immunosorbent assay (ELISA) kits from R&D Systems, Inc. (Minneapolis, MN, USA).

### 2.8. Statistical Analyses

Data are expressed as the mean ± standard error. Statistical analyses were performed using JMP Pro 14 software (SAS Institute Inc., Cary, NC, USA). The sample size was chosen based on comparable experiments from our previous experiments. Briefly, in one-way analysis of variance study, sample sizes of 10 and 10 were obtained from the 2 groups to compare the means. The total sample of 20 subjects achieves 100% power for detecting differences among the means versus the alternative of equal means using an F test with a 0.05 significance level. The size of the variation in the means was represented by their standard deviation, which was 0.77. The common standard deviation within a group was assumed to be 1.00. We used PASS 14.0.9 (PASS Software by NCSS, LLC, Kaysville, UT, USA) to compute the statistical power. Statistical comparisons between two different groups were made using a *t*-test, and the differences between three groups were examined using a one-factor analysis of variance (ANOVA) followed by Turkey’s HSD test. Probability values of *p* < 0.05 were considered statistically significant.

## 3. Results

### 3.1. Animals Welfare

There was no statistical difference in the death rate between the exosome group and the PBS group. However, during the experimental session, three rats in the exosome group and two rats in the PBS group were euthanized due to excessive weight loss.

### 3.2. Exosome Characterization

**Figure 1:**
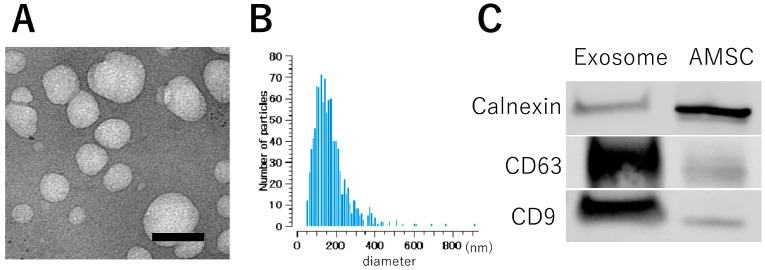
Characteristics of exosome derived from AMSC. (**A**) Electron microscopy depicts exosomes with a rounded morphology and an approximate diameter of 100 nm. (**B**) Size distribution, ascertained by the nanoparticle analyzer, delineates exosome dimensions ranging from 70–400 nm, which showed 110 nm peak. (**C**) Western blot detection shows heightened expression of CD9 and CD63 in the exosome fraction, while a reduced signal is observed for Calnexin compared to the AMSC sample. Bar: 100 nm.

TEM images demonstrated a consistent morphology of the exosomes, presenting an average diameter of approximately 100 nm. (Figure 1A) This observation was further confirmed by the size distribution profiles derived from the nanoparticle analyzer, illustrating a range between 70 and 400 nm with a peak at 110 nm (Figure 1B). Western blot analysis demonstrated elevated expression levels of CD9 and CD63, recognized as specific markers for exosomes, in comparison to the AMSC suspension. Conversely, the expression of calnexin, a marker with low expression in exosomes, exhibited a diminished profile in the exosome (Figure 1C). These results conclusively confirm the presence of exosomes in the AMSC-derived samples.

### 3.3. Intranasal Exosome Administration Ameliorates Short- and Long-Term Memory Impairment

**Figure 2:**
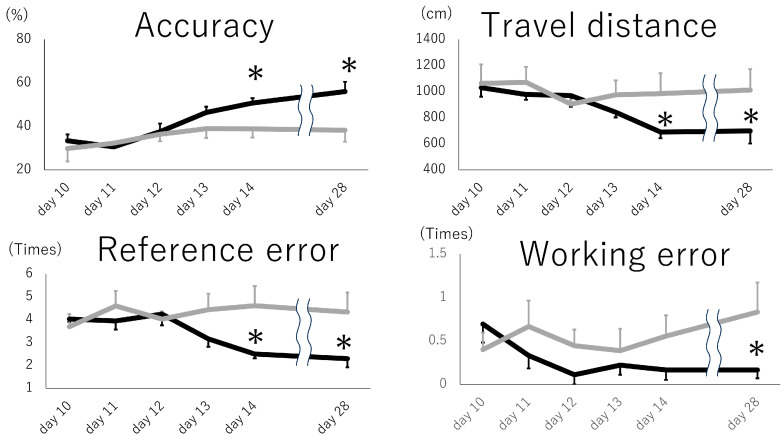
Short- and long-term memory impairment test. The animals that received intranasal exosomes exhibited better memory function in accuracy (correct arm entry/all arm entry), travel distance, reference error (wrong arm entry), and working error (entry into arms where pellets were previously collected). Black: exosome group, gray: PBS group, *: *p* < 0.05.

Memory tasks using the eight-arm maze revealed that animals receiving intranasal exosomes demonstrated better accuracy (correct arm entry/all arm entry) compared to those receiving PBS (Figure 2). These differences were evident on day 5, the last day of the five consecutive trials, and persisted up to 14 days after the last trial (day 28). Similar trends were observed in travel distance, where the exosome group exhibited shorter distances traveled to obtain the pellets, and in reference errors, indicating fewer entries into the wrong arm. These differences were also notable on day 28. Working errors, representing re-entry into previously explored arms, were significantly reduced in the exosome group on day 28. These results suggest that exosome administration improved both short- and long-term memory impairment.

### 3.4. Biodistribution of Exosome

**Figure 3:**
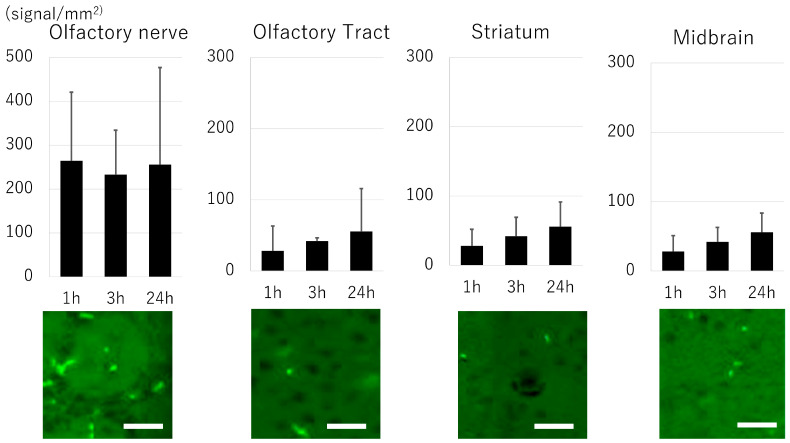
Biodistribution of exosome. A large number of exosomal signals (approx. 250 signals/mm^2^) can be found in the olfactory nerve starting from one hour after transplantation. The signal can also be found in the olfactory tract, striatum, and midbrain. Note that the *Y* axis is different between the olfactory nerve and others. The representative figure shows the signal at each area 24 h after exosome administration (bar = 20 μm).

The distribution of exosomes at the olfactory nerve, olfactory bulb, striatum, and midbrain was evaluated using fluorescence imaging (Figure 3). Exosomes were abundantly observed (approx. 250 signals/mm^2^) in the olfactory nerve 1 h after administration. Although the number of positive signals was smaller compared to the olfactory nerve, positive signals were evident in the olfactory tract, striatum, and midbrain, and the signal in these areas showed a gradual increase up to 24 h after transplantation.

### 3.5. Exosome Ameliorate Neuronal Damage in CA1

**Figure 4:**
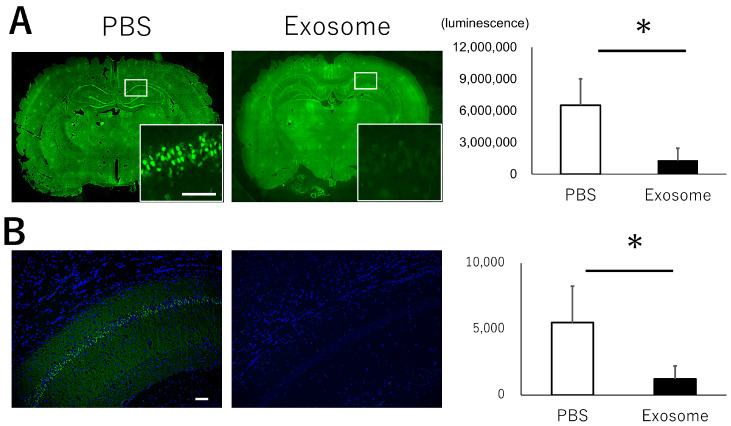
Neural damage in hippocampus. (**A**) Apoptosis in hippocampus was evaluated in CA1 lesion using TUNEL staining at day 3. Apoptotic cells were significantly decreased in the exosome group compared with that of PBS group. (**B**) Neural degeneration was observed in CA1 lesion using Fluoro-Jade C staining at day 7. Exosome group exhibited significantly less degenerated neural cells compared with PBS group. Bar = 100 μm, *: *p* < 0.05.

**Figure 5:**
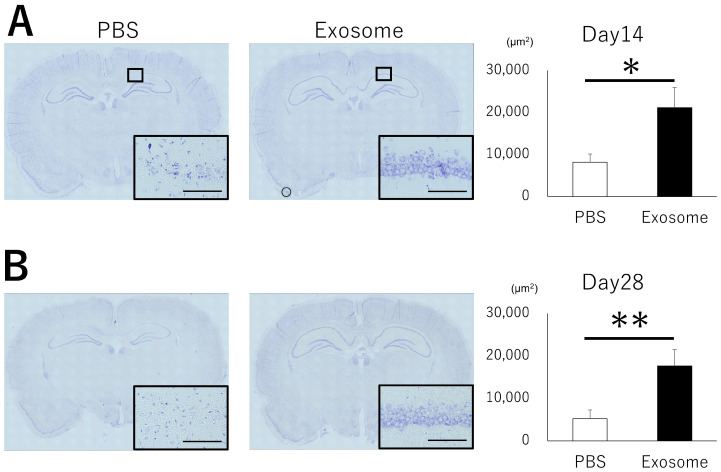
Neural survival in hippocampus. Neural cells were evaluated with Nissl staining. Nuclear aggregation and structural alterations were observed in the PBS group, whereas these were preserved in the exosome group at both day 14 (**A**) and day 28 (**B**). Bar = 100 μm, *: *p* < 0.05, **: *p* < 0.01.

The hippocampus, particularly the CA1 area, is known to play a critical role in memory impairment and is frequently affected by HIBI. To investigate the functional mechanisms of intranasal exosome administration, we evaluated neuronal apoptosis and neural degeneration. While the CA1 region exhibited abundant apoptotic cells (Figure 4A) and degenerated cells (Figure 4B) in the PBS group, exosomes successfully reduced the presence of apoptosis and neural degeneration. These findings were further confirmed by the survival of neural cells in the CA1 region. Nissl staining revealed that cells exhibited aggregated nuclei and structural alterations in the PBS group, whereas normal morphologies were preserved in the exosome group. The observations were evident not only in the acute phase but also in the chronic phase observed at days 14 and 28 (Figure 5). These results indicate that exosome administration rescued cell survival in the hippocampus, leading to better preservation of memory function.

### 3.6. Microglial Inflammation Were Inhibited by Intranasal Exosome Administration

**Figure 6:**
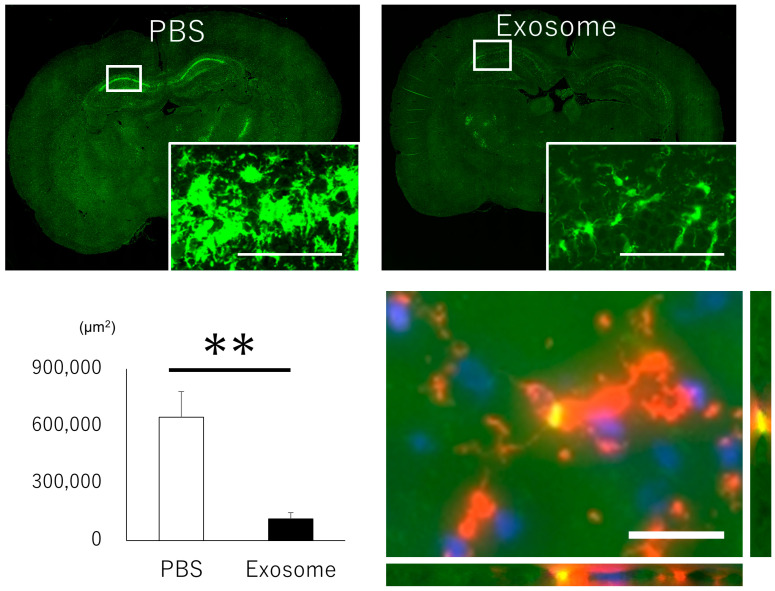
Microglial activation in hippocampus. Activated microglia were prominently observed in the PBS group, characterized by cell proliferation and a change in morphology to an amoeboid shape. In contrast, these changes were notably attenuated in the exosome-treated group (upper and left lower panels) Bar = 100 μm. Exosomes labeled with ExoSparkler (green) were found to co-localize with microglia (orange), suggesting the anti-inflammatory role of exosomes (Nuclear staning by DAPI. right lower panel). Bar = 20 μm, **: *p* < 0.01.

Given that brain inflammation is a pivotal component of neural injury, we examined microglial activation in the hippocampus. Analysis of activated microglia via Iba1 staining revealed proliferation and transformation into amoeboid shapes of microglia in the PBS group, whereas the exosome group effectively suppressed this activation (Figure 6, upper and left lower panels). Furthermore, labeled exosomes were found to co-localize with microglia, indicating the anti-inflammatory role of exosomes (Figure 6, right lower panel).

**Figure 7:**
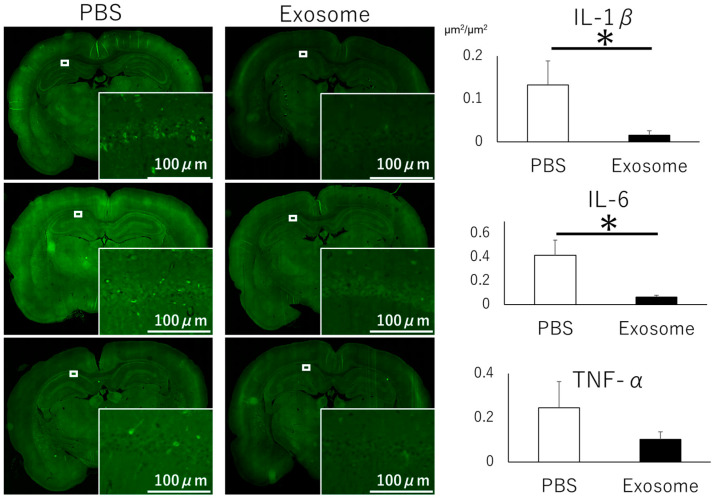
The exosome-treated group exhibited lower cytokine expression compared to the PBS-treated group. Levels of IL-1b (upper panel), IL-6 (middle panel), and TNF-α (lower panel) are depicted. Bar = 100 μm, *: *p* < 0.05.

To further investigate the anti-inflammatory effects of exosomes, we evaluated inflammatory cytokines. That rats that received intranasal PBS exhibited lower levels of IL-1β and IL-6 expression in the hippocampus, and a higher trend in TNF-α expression compared with those that received intranasal exosomes (Figure 7). These findings demonstrate that intranasal administration of AMSC-derived exosomes alleviated microglial inflammation.

### 3.7. Inhibitory Role of Exosome for Microglial Activation

**Figure 8:**
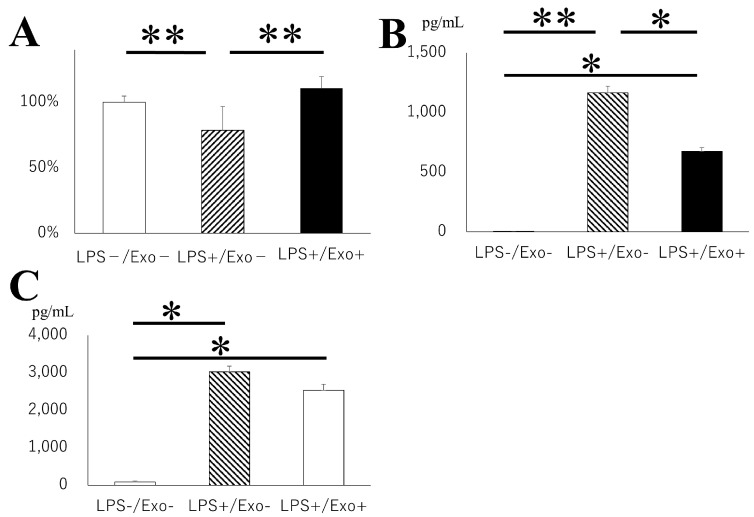
In vitro analysis of anti-inflammatory effect of exosomes. Exosomes were administered into mouse BV2 cell. Cell viability (**A**), IL-6 production (**B**), and TNF-α production (**C**) were assessed between LPS group and LPS with exosome group. *: *p* < 0.05, **: *p* < 0.01.

The anti-inflammatory effects of exosomes against microglia are further evaluated in an in vitro model using mouse microglial BV2 cells. The administration of LPS significantly reduced microglial viability compared with the control group, whereas exosomes notably reversed the viability (Figure 8A). Furthermore, IL-6 production was significantly upregulated with LPS stimulation, where exosome administration successfully reversed the expression. (Figure 8B) Similar trends were observed for TNF-α (Figure 8C). These data clearly demonstrate the immunosuppressive capability of exosomes.

## 4. Discussion

In this study, the authors have demonstrated that intranasally transplanted exosomes derived from AMSC can migrate into the brain as early as one hour and continuously increased up to 24 h after transplantation, and successfully mitigated microglial inflammation and neural damage, resulting in the restoration of cognitive function.

Cognitive functional impairment including short- and long-term memory disturbance is the most critical sequelae for patients who survive HIBI, and damage in the CA1 area of the hippocampus is considered to be responsible for these symptoms [23,24,25,26,27,28,29]. In addition to the fragility of the CA1 neuronal cells to ischemic insult as a consequence of acute damage, recent evidence shows that microglial inflammatory reactions can also contribute to delayed neuronal cell death, which may take 2–3 days after the initial insult [30]. Thus, delayed neuronal cell death in the hippocampus has been the rescue target to ameliorate neurocognitive function. Neural cell transplantation of progenitor cells into the hippocampus and neurogenesis directed by reprograming factors Klf4, Sox2, Oct4, c-Myc successfully achieved cognitive function recovery, which highlights the importance of hippocampus repair in cognitive function [31,32,33].

MSC-derived exosomes have successfully attenuated hippocampal injury. Multiple therapeutic mechanisms have been reported, including anti-apoptotic function, astrocytic/microglial deactivation, preserving blood–brain barrier integrity, neurogenesis, and synaptogenesis. Among them, microglial deactivation has been a subject of intense scrutiny. Chen et al. reported that Circular RNA SCMH1 encapsuled in adipose-derived MSC achieved M2 polarization of microglia, whereas others reported that circulating RNA ribosomal protein S5 (circ-Rps5) or microRNA-233-3p were responsible for M2 polarization [34,35,36]. These data were in line with our results that exosomes possess the potential to recover hippocampal neuronal cells and mitigate cognitive damage. Other mechanisms are also reported. Chen et al. showed that intravenous exosome transplantation achieved neurogenesis and neuritogenesis through 2′, 3′-Cyclic nucleotide 3′-phosphodiesterase-encapsuled exosomes, and Nakano et al. demonstrated that mitochondria morphological abnormality in astrocytes and neurons were improved by exosome transplantation, resulting in cognitive recovery [33,37]. Although, further evaluation is necessary to fully elucidate the precise molecular mechanisms of exosomes, their recovery potential for hippocampal injury is worth investigating.

Several transplantation routes have been explored for delivering exosomes to the brain. Intravenous transplantation offers the advantage of less invasive procedures compared to intracerebral/intrathecal transplantation. However, systemic dilution presents a significant challenge with this approach, as most exosomes transplanted intravenously accumulate in the liver, lung, and spleen, resulting in only approximately 0.00–0.01% reaching the brain [9]. Consequently, intranasal administration is increasingly recognized as a promising therapeutic route for delivery [38,39]. The method has been reported to be superior to intravenous transplantation, as none of the transplanted exosomes are found in the brain 24 h after intravenous transplantation. In contrast, significantly larger amounts of exosomes can be found in the brain through intranasal transplantation [40]. Exosomes are small enough to be absorbed into the olfactory bulb and trigeminal nerve when transplanted into the nasal cavity. Two different forms of transport, intracellular and extracellular, are reported to be involved in intranasal delivery [41]. Intracellular transport involves endocytosis into the olfactory sensory neurons of olfactory or trigeminal neurons, followed by intraneuronal transport along the axon through the cribriform plate to the olfactory bulb or directly to the pons. A previous report demonstrated that the majority of PKH-labeled MSC-derived exosomes delivered intranasally to a mouse model of Alzheimer’s were accumulated in the layer of immature olfactory sensory neurons. The exosomes were further found to be transferred through the cribriform plate, concentrated in the glomerulus, and then progressed to the mitral cells, subsequently being transported into deeper brain areas [42]. The extracellular pathway involves the perivascular transport system. Exosomes enter the systemic circulation in the lamina propria through the nasal blood vessels, or they may enter the cerebrospinal fluid through olfactory ensheathing cells, subsequently reaching blood vessels in the CNS. The capacity of exosomes to traverse the blood–brain barrier enables them to reach inflammatory areas through receptor-mediated transcellular transport or endocytosis. Consistent with these theories, we found a large amount of labeled exosomes in the olfactory bulb. We further found that exosomes subsequently migrate into the brain parenchyma during the course of time, which is in line with another report in which Betzer et al. utilized gold nanoparticles to track intranasally administered exosomes, and found that the number of particles continuously increases up to 24 h after transplantation, mostly accumulating around the inflammation site [40]. Given the information that exosome’s homing mechanism is inflammatory-driven, this method can be ideal to mitigate acute brain disease causing inflammation [43]. Intranasal exosome administration has been reported to be effective in various neurological disease models, including ischemic stroke, traumatic brain injury, perinatal brain injury, neurodegenerative disease, and psychiatric disease [11]. Furthermore, three clinical trials are currently on-going using MSC-derived exosomes through intranasal administration: epilepsy (NCT: 05886205), Alzheimer’s disease (NCS04388982), and preterm birth (NCT: 05490173).

The method for precisely tracking exosome distribution in the brain is of paramount importance. In this study, the authors utilized a commercially available exosome labeling kit. Although, PHK lipophilic dye is commonly employed to observe and track the distribution of exosomes in the brain due to its high fluorescence and stability achieved by intercalating an aliphatic region into the exposed lipid bilayer, these dyes may form nanoparticles that could lead to false-positive signals or alter the size of EVs, thereby affecting cellular uptake and biodistribution [44,45]. The dyes used in this study are reported to show less aggregation and have minimal impact on exosome properties, enabling more accurate observation of exosome dynamics [46].

There are several limitations to be considered in this manuscript. First, the precise number of exosomes transferred into the brain was not quantitated. We labeled the exosomes with commercially available fluorescent dye which has a short wave length for penetrating the skull. Although the fluorescence signaling can be compared between the sections, the total number of exosomes cannot be calculated. Isotope labeling may be required to elucidate the precise absorbance rate of exosomes. Second, we were not able to show the contents in the exosomes which attenuated microglial activation, as miRNAs, such as mir760-3p, 342-3p, 330-3p, are reported to contribute to microglial deactivation [47,48,49]. Our previous report also denoted that mir125a-3p showed an anti-inflammatory effect through the deactivation of neutrophil [50]. Microarray followed by real-time PCR is necessary to evaluate the therapeutic contents. Third, we were not able to track neurological impairment for longer than 28 days. It is important to monitor this for a longer time to gain a better understanding of functional recovery.

## 5. Conclusions

Intranasal administration of mesenchymal stem cell-derived exosomes alleviates hypoxic-ischemic brain injury through an anti-inflammatory effect, and can be one unique treatment method.

## Data Availability

The data can be transferred from the corresponding author, M.K., upon reasonable request.
